# Targeted Stimulation of an Orbitofrontal Network Disrupts Decisions Based on Inferred, Not Experienced Outcomes

**DOI:** 10.1523/JNEUROSCI.1680-20.2020

**Published:** 2020-11-04

**Authors:** Fang Wang, James D. Howard, Joel L. Voss, Geoffrey Schoenbaum, Thorsten Kahnt

**Affiliations:** ^1^Department of Neurology, Feinberg School of Medicine, Northwestern University, Chicago, Illinois 60611; ^2^Department of Medical Social Sciences, Feinberg School of Medicine, Northwestern University, Chicago, Illinois 60611; ^3^Department of Psychiatry and Behavioral Sciences, Feinberg School of Medicine, Northwestern University, Chicago, Illinois 60611; ^4^National Institutes on Drug Abuse, Intramural Research Program, Baltimore, Maryland 21224; ^5^Department of Psychology, Weinberg College of Arts and Sciences, Northwestern University, Evanston, Illinois 60208

**Keywords:** decision-making, model based, model free, orbitofrontal cortex, sensory preconditioning, transcranial magnetic stimulation

## Abstract

When direct experience is unavailable, animals and humans can imagine or infer the future to guide decisions. Behavior based on direct experience versus inference may recruit partially distinct brain circuits. In rodents, the orbitofrontal cortex (OFC) contains neural signatures of inferred outcomes, and OFC is necessary for behavior that requires inference but not for responding driven by direct experience. In humans, OFC activity is also correlated with inferred outcomes, but it is unclear whether OFC activity is required for inference-based behavior. To test this, we used noninvasive network-based continuous theta burst stimulation (cTBS) in human subjects (male and female) to target lateral OFC networks in the context of a sensory preconditioning task that was designed to isolate inference-based behavior from responding that can be based on direct experience alone. We show that, relative to sham, cTBS targeting this network impairs reward-related behavior in conditions in which outcome expectations have to be mentally inferred. In contrast, OFC-targeted stimulation does not impair behavior that can be based on previously experienced stimulus–outcome associations. These findings suggest that activity in the targeted OFC network supports decision-making when outcomes have to be mentally simulated, providing converging cross-species evidence for a critical role of OFC in model-based but not model-free control of behavior.

**SIGNIFICANCE STATEMENT** It is widely accepted that the orbitofrontal cortex (OFC) is important for decision-making. However, it is less clear how exactly this region contributes to behavior. Here we test the hypothesis that the human OFC is only required for decision-making when future outcomes have to be mentally simulated, but not when direct experience with stimulus–outcome associations is available. We show that targeting OFC network activity in humans using network-based continuous theta burst stimulation selectively impairs behavior that requires inference but does not affect responding that can be based solely on direct experience. These results are in line with previous findings in animals and suggest a critical role for human OFC in model-based but not model-free behavior.

## Introduction

Many decisions are made based on expectations about their likely outcomes. Such expectations can reflect what we have experienced in the past, for instance, when ordering your favorite dish at a familiar restaurant. For many other decisions in life, such as deciding to try out a new restaurant or enrolling in a PhD program, direct experience is lacking, and outcome expectations need to be mentally simulated or inferred.

Expectations arising from these two different origins, which may compete for control over behavior (Daw et al., 2005; Lee et al., 2014), are thought to recruit partially distinct brain circuits (Balleine and Dickinson, 1998; Daw et al., 2011; O'Doherty et al., 2017). Whereas much research has focused on behavior that is based on direct experience (Schultz, 1998; Tricomi et al., 2009; Wunderlich et al., 2012), less is known about the neural representations that support behavior based on inferred outcomes, particularly in humans.

Work across animal species suggests that the orbitofrontal cortex (OFC), together with the hippocampus, is important for behavior based on inference (Rudebeck and Murray, 2014; Wikenheiser and Schoenbaum, 2016). For instance, in tasks that require mental simulation, neural activity in the rodent OFC represents inferred outcomes in almost the same way as it signals directly experienced outcomes (Takahashi et al., 2013; Sadacca et al., 2018). Interestingly, however, the rat OFC is not required for behavior based on directly experienced outcomes, but it is only necessary when responding requires inference (Jones et al., 2012; Takahashi et al., 2013). This suggests that rodent OFC is selectively required for the simulation of outcomes. Recent work in humans has shown similar neural correlates of inferred outcomes in the OFC (Barron et al., 2013; Wang et al., 2020), but whether human OFC networks are required for behavior based on such inferred outcomes is unclear.

Causal studies on human OFC function have traditionally been limited to naturally occurring lesions (Reber et al., 2017; Vaidya et al., 2019). However, we have recently developed a novel network-based transcranial magnetic stimulation (TMS) approach to noninvasively target activity in human OFC networks (Howard et al., 2020). Similar to previous work targeting the hippocampal network (Wang et al., 2014), this approach uses resting-state functional magnetic resonance imaging (rsfMRI) to individually define stimulation coordinates in the lateral prefrontal cortex (LPFC) that are part of the central–lateral OFC network (Kahnt et al., 2012; Zald et al., 2014). We have recently shown that this targeted TMS protocol selectively affects connectivity in lateral OFC networks, in parallel with disrupting OFC-dependent behavior (Howard et al., 2020).

**Figure 1.**
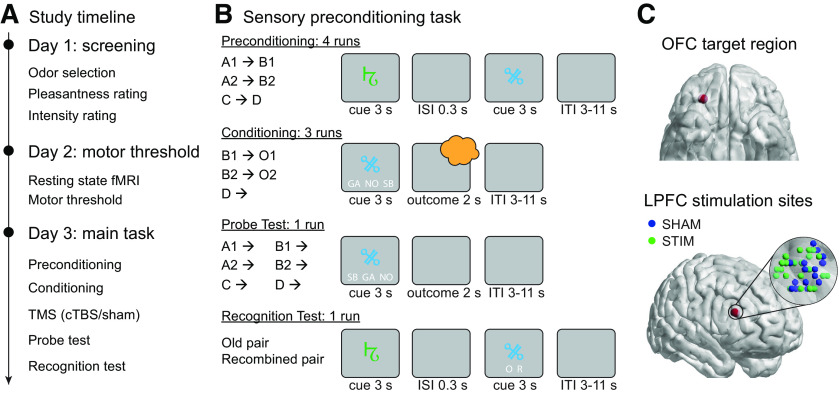
Experimental design and sensory preconditioning task. ***A***, Experimental timeline. ***B***, Participants learned cue pairs during preconditioning (A → B, C → D). During conditioning, they learned associations between the second cue in each pair and one of two food odors (O1 or O2) or odorless air (B → odor reward, D → odorless air). During the probe test, participants were asked to make outcome predictions to all cues, but no outcomes were delivered. Finally, subjects completed a recognition task testing for memory of cue–cue associations. ***C***, Top, Targeted area in central-lateral OFC (*x* = 28, *y* = 38, *z* = −16). Bottom, Individual stimulation sites in LPFC, overlaid on a glass brain using BrainNet Viewer (Xia et al., 2013). For each participant, we identified a coordinate within an 8 mm LPFC sphere (centered on *x* = 48, *y* = 38, *z* = 20) that showed maximal rsfMRI connectivity with the intended OFC target. We stimulated these individually determined LPFC coordinates using infrared MRI-guided stereotactic neuronavigation.

In the current study (Fig. 1*A*), we applied this novel OFC-targeted brain stimulation approach in the context of a sensory preconditioning task that was designed to isolate inference-based behavior from responding that can be based on direct experience (Jones et al., 2012; Wimmer and Shohamy, 2012; Wang et al., 2020). This task consists of three phases (Fig. 1*B*). First, during preconditioning, pairs of sensory cues are repeatedly presented (A → B, C → D). Next, during conditioning, the second cue of each pair is associated with reward and no reward, respectively (B → reward, D → no reward). During the final probe test, reward-related responding to each cue (A, B, C, and D) is separately probed under extinction conditions. Reward-related responses to cue A indicate that subjects step through the associations A → B and B → reward to infer A → reward. In contrast, such responses to cue B do not require inference because direct experience with the cue–outcome pairing is available. We predicted that disrupting OFC network activity with OFC-targeted TMS will impair inference-based behavior (responding to cue A), but not behaviors that can be based entirely on direct experience alone (responding to cue B).

## Materials and Methods

### 

#### Subjects

In total, 71 healthy adults participated in a screening session. Of these, 52 passed screening, were randomly assigned to the sham (SHAM group: *n* = 25; 13 female) or stimulation (STIM) group (STIM group: *n* = 27; 15 female), and participated in the experiment. All participants provided written informed consent to participate and were compensated with $20/h for behavioral testing and $40/h for TMS and MRI scanning. The study protocol was approved by the Northwestern University Institutional Review Board. One participant in the STIM group withdrew during the experiment. Data from four participants (two per group) were excluded from all analyses because their performance in the last run of conditioning was not significantly above chance (*p* > 0.05, binomial test). This left a total of 47 participants (SHAM group: *n* = 23; 12 female; mean age, 25.24 ± 0.86 years (mean ± SEM); STIM group: *n* = 24; 13 female; mean age, 25.30 ± 0.70 years) from whom data were analyzed. Of those, data from four participants (one SHAM, three STIM) from the recognition memory test of the experiment were not recorded because of technical problems.

#### Stimuli and odor delivery

Visual cues consisted of 14 abstract symbols, and 12 of them were randomly grouped into six pairs for each participant. Two pairs served as A1–B1 pairs, two served as A2–B2 pairs, and two served as C–D pairs. The two remaining symbols were used to form two catch–trial pairs (E–E) in which the same symbols were presented twice in a row (i.e., E1–E1, E2–E2). The two symbols constituting a pair were presented in different colors (e.g., first symbol blue, second symbol green; counterbalanced across participants).

As in our previous studies, the current experiment used food odors as reward in hungry participants (Howard et al., 2015, 2020; Howard and Kahnt, 2017, 2018; Suarez et al., 2019). Eight food odors (four sweet: strawberry, caramel, gingerbread, and yellow cake; four savory: potato chip, pot roast, garlic, and pizza) were provided by Kerry and International Flavors and Fragrances. Odors were delivered to participants' nose using a custom-built and computer-controlled olfactometer (Howard et al., 2020; Wang et al., 2020). The olfactometer was equipped with two independent mass flow controllers (Alicat), which allow dilution of any given odorant with odorless air. Odorless air was delivered constantly during the experiment, and odorized air was mixed into the airstream at specific time points. The overall flow rate was kept constant at 3.2 L/min throughout the task, such that odor delivery did not involve a change in overall airflow or any noticeable change in somatosensory stimulation.

#### Experimental design and behavioral task

The study was conducted over 3 d (Fig. 1*A*) and included (1) a screening session, (2) an MRI and TMS motor threshold (MT) session, and (3) a main task session. The MRI and TMS MT session was conducted on average 18 d (SEM, 4.16) after the screening session. And the average delay between the TMS MT and main task sessions was 4 d (SEM, 0.94). Participants were instructed to arrive in a hungry state (fast for at least 4 h) for the screening and main task sessions.

##### Screening session

After informed consent and screening for eligibility, participants rated the pleasantness of eight food odors. In each trial, they were presented with one of the eight food odors for 2 s and were instructed to make a medium-sized sniff. They then rated the pleasantness of the delivered odor on a scale from “most disliked sensation” to “most liked sensation.” Each food odor was presented three times in randomized order, and ratings were averaged. We then selected one sweet and one savory odor that were both rated as pleasant (i.e., pleasantness above neutral) and were as closely matched as possible. The two selected odors were then used as a reward for that individual participant in the main task session. If no such two odors were found, participants were excluded from further participation in the study. Next, participants rated the intensity and pleasantness of the two selected odors as well as odorless air. The scale of the intensity rating was from “undetectable” to “strongest sensation imaginable.”

##### MRI and TMS motor threshold session

We acquired a T1-weighted structural MRI scan for the purpose of TMS neuronavigation and an 8.5 min rsfMRI scan for individually defining OFC-targeted stimulation coordinates (see below). We then measured resting MT by delivering single TMS pulses over left motor cortex. MT was defined as the minimum percentage of stimulator output necessary to evoke 5 visible thumb movements in 10 stimulations.

##### Main task session

The main task session consisted of preconditioning, conditioning, TMS, probe test, and a cue–cue recognition memory test (Fig. 1*B*). In four preconditioning runs, participants were instructed to learn the associations between the two cues in each pair [A → B (A1 → B1, A2 → B2), C → D (C1 → D1, C2 → D2), E → E (E1 → E1, E2 → E2)]. The cues in a given pair were presented one after another for 3 s each, separated by an interstimulus interval of 300 ms. A fixation cross appeared between trials for a variable intertrial interval (ITI) between 3 and 11 s. To ensure attention to the cue pairs, participants were instructed to memorize the cue pairs, press a button if the second cue was different from the first cue, and withhold a response if the two cues were identical. To facilitate learning, in the first two runs of preconditioning, each cue pair was repeated three times in a row. In the remaining preconditioning runs, the order of cue pairs was randomized across trials.

Next, participants performed three runs of conditioning, during which the second cue of each pair [cues B (B1, B2) and D (D1, D2)] was presented individually for 3 s. Participants were instructed to indicate by button press which outcome [e.g., strawberry odor (SB), garlic odor (GA), or no odor (NO)] they expected following the cue. If they expected strawberry, they were asked to select “SB”; if they expected garlic, they were asked to select “GA”; if they expected no odor, they were asked to select “NO.” Participants made their prediction by pressing a button with the index, middle, or ring fingers of their right hand corresponding to the positions of SB, GA. and NO on the screen. The positions of the abbreviated names were randomized across trials. Irrespective of their selection, the outcome was presented for 2 s immediately after the cue. However, “too slow” was displayed if participants failed to respond within 3 s. Each cue–outcome association was repeated four times in each run in pseudorandomized order.

After the conditioning phase, participants received OFC-targeted continuous theta burst stimulation (cTBS; see below). The probe test followed immediately after the stimulation. In each trial of the probe test, cue A (A1, A2), B (B1, B2), C (C1, C2), or D (D1, D2) was presented individually under extinction conditions (odorless air was delivered throughout) to prevent further learning. Each cue was presented four times in pseudorandomized order. Participants were instructed to predict the outcome after each cue, as they did during the conditioning phase. They were further instructed to use the cue–cue associations learned in the first phase to infer the outcomes associated with the preconditioned cues (Wang et al., 2020). The durations of cue presentation and the ITI were the same as during the conditioning phase.

Following the probe test, participants were tested for their memory of the cue–cue associations in a recognition task. On each trial, participants were presented with either an original cue pair or with a newly recombined pair (i.e., consisting of cues belonging to different pairs). Pairs were presented sequentially as during preconditioning, and participants were asked to indicate using a button press whether a pair was old (O) or recombined (R) after the second cue was presented.

#### MRI data acquisition

MRI data were acquired at the Northwestern University Center for Translational Imaging using a Siemens 3 T PRISMA system equipped with a 64-channel head coil. rsfMRI scans were acquired with an echoplanar imaging (EPI) sequence with the following parameters: repetition time (TR), 2 s; echo time (TE), 22 ms; flip angle, 90°; slice thickness, 2 mm, no gap; number of slices, 58; interleaved slice acquisition order; matrix size, 104 × 96 voxels; field of view, 208 × 192 mm; multiband factor, 2. To minimize susceptibility artifacts in the OFC, the acquisition plane was tilted ∼25° from the anterior commissure–posterior commissure line. The rsfMRI scan consisted of 250 EPI volumes covering all but the most dorsal portion of the parietal lobes. In addition, a 3D 1 mm isotropic T1-weighted structural scan was also collected (TR, 2300 ms; TE, 2.94 ms; flip angle, 9°; field of view, 176 × 256 × 256 mm)

#### fMRI data preprocessing

Preprocessing of functional images was performed using Statistical Parametric Mapping (SPM12; https://www.fil.ion.ucl.ac.uk/spm/). To correct for head motion during scanning, all rsfMRI images were aligned to the first acquired image. The mean realigned images were then coregistered to the anatomic image, and the resulting registration parameters were applied to all realigned EPI images. Finally, coregistered EPI images were resliced and smoothed with a 6 × 6 × 6 mm Gaussian kernel. To generate forward and inverse deformation fields, the anatomic image was normalized to Montreal Neurologic Institute (MNI) space using the six-tissue probability map provided by SPM12.

#### OFC-targeted TMS

We used our previously established network-based OFC-targeted TMS protocol (Howard et al., 2020). TMS was delivered using a MagPro X100 stimulator connected to a MagPro Cool-B65 butterfly coil (MagVenture). We used a cTBS protocol involving a 40 s train of three-pulse 50 Hz bursts delivered every 200 ms (5 Hz, totaling 600 pulses), and stimulation was delivered at an intensity of 80% MT in the STIM group and 5% MT in the SHAM group. Previous work has shown that 40 s of this cTBS protocol at 80% MT has inhibitory aftereffects that last for 50–60 min over motor cortex (Huang et al., 2005). We applied stimulation at 5% MT as a sham control because TMS at low intensities (0−10% MT) is not expected to have any neural effects (Wang et al., 2014; Hermiller et al., 2019; Hebscher and Voss, 2020). As in our previous study (Howard et al., 2020), the target coordinate was defined as a location in the right LPFC that showed maximal functional connectivity with the right OFC seed coordinate (see details below). The orientation of the coil tilted was such that the long axis of the figure-of-eight coil was approximately parallel to the long axis of the middle frontal gyrus. All participants were informed that they may experience muscle twitches in the forehead, eye area, and jaw during stimulation. We delivered two single test pulses to test for tolerability before cTBS was delivered. Immediately after the last pulse of cTBS, the time was noted. All subsequent testing (probe test and recognition memory) took place within 33 ± 1.92 min of the end of TMS, and this time did not differ between groups (*t* = 0.24, *p* = 0.814).

#### Coordinate selection for OFC-targeted TMS

Stimulation coordinates on the right LPFC were determined for each individual participant based on rsfMRI connectivity with a right central-lateral OFC seed region using a previously described procedure (Fig. 1*C*; Howard et al., 2020). We targeted the right central-lateral OFC because activity in this region has previously been shown to correlate with outcome-specific expectations (Klein-Flügge et al., 2013; Howard et al., 2015; Howard and Kahnt, 2017), but we do not propose a lateralization of OFC function and assume that bilateral OFC-targeted stimulation would yield even larger effect sizes. Briefly, we first created two spherical masks of 8 mm radius around an LPFC coordinate (*x* = 48, *y* = 38, *z* = 20) and an OFC seed coordinate (*x* = 28, *y* = 38, *z* = −16) in MNI space, both inclusively masked by the gray matter tissue probability map provided by SPM12 (thresholded at >0.1). These masks were then inverse normalized to each participant's native space using the inverse deformation field generated by normalizing the anatomic image. We then estimated a general linear model with the average rsfMRI time series in the OFC mask as the regressor of interest and realignment parameters as regressors of no interest. The voxel in the LPFC mask that had the highest functional connectivity with the OFC seed was defined as a stimulation coordinate. We used infrared MRI-guided stereotactic neuronavigation (LOCALITE) to apply stimulation to this coordinate.

#### Statistical analysis

The main statistical analyses were conducted based on a total of 47 subjects in the two groups (SHAM group: *n* = 23, 12 female; STIM group: *n* = 24, 13 female). Simple between-group effects were tested using unpaired *t* tests. Results from parametric tests were confirmed using permutation tests involving 10,000 random group assignments. Interactions were tested using R (R Core Team, 2018) and the lme4 package (Bates et al., 2012). Specifically, we performed a linear mixed-effect analysis on odor pleasantness ratings with group (SHAM vs STIM group) and odor (odor vs odorless) as independent variables. In addition, to test the interaction among group, cue type, and time on reward predictions during conditioning, we used a generalized linear mixed model with group (SHAM vs STIM group), cue (B vs D), and time (three runs) as independent variables. Finally, the interaction between group and cue type on reward predictions during the probe test was tested using a generalized linear mixed model with group (SHAM vs STIM group) and cue type (A vs B) as independent variables. Effects of group and cue type (and their interaction) on response times were also tested using linear mixed models. In all analyses, subjects were modeled as random intercept effects. There were no obvious deviations from normality or homoscedasticity based on visual inspection of residual plots. We computed *p* values by likelihood ratio tests (χ^2^) of the full model including the effect of interest against the reduced model without the effect of interest. Statistical thresholds were set to *p* < 0.05, two-tailed unless indicated otherwise.

## Results

### Odor ratings and learning performance

The experiment took place across 3 d (Fig. 1*A*). Days 1 and 2 consisted of a screening visit and an MRI session (anatomical and rsfMRI), respectively. Day 3 involved a sensory preconditioning task and network-based OFC-targeted TMS. On day 3, subjects (SHAM group, *n* = 23; STIM group, *n* = 24) in both groups arrived fasted (they had not eaten for 11 ± 4.27 h; group difference, *t*_(45)_ = 1.00, *p* = 0.321) and with similar levels of hunger (*t*_(45)_ = 1.28, *p* = 0.205). Subjects first learned associations between pairs of abstract visual cues during preconditioning (A → B, C → D; Fig. 1*B*). Next, they learned that a pleasant food odor followed cue B, whereas cue D was always followed by odorless air (Fig. 1*B*). To measure reward expectations, participants were asked to predict the outcome associated with the presented cue via button press.

**Figure 2.**
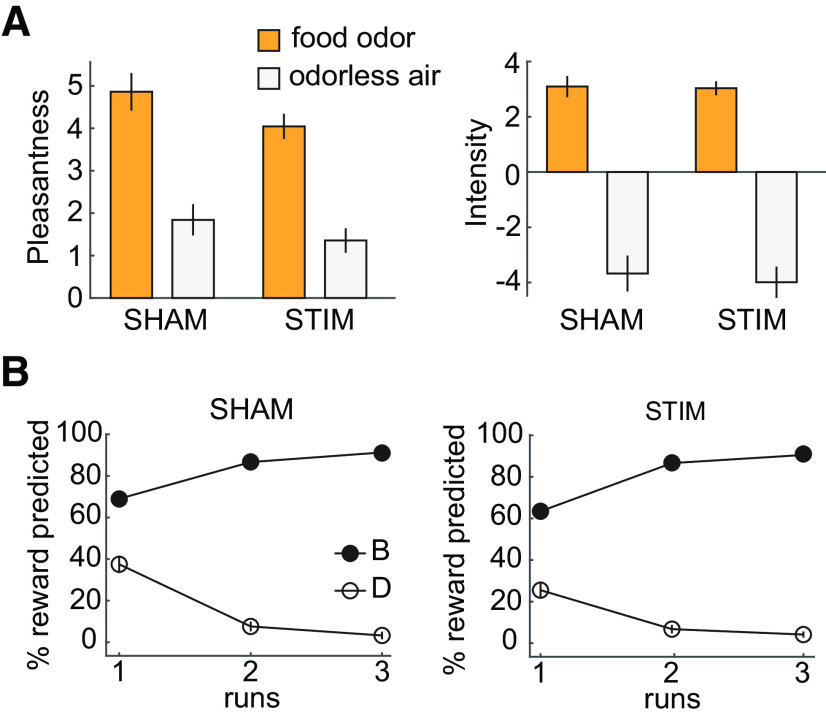
Odor ratings and behavioral performance during conditioning. ***A***, Participants rated the pleasantness (left) and intensity (right) of food odors significantly higher than odorless air (*p* < 0.001), but ratings did not differ between groups (*p* values > 0.14). ***B***, The percentage of trials in which an odor reward was expected after cue B increased relative to cue D across time during conditioning, and there were no group differences. Error bars depict SEM (SHAM group, *n* = 23; STIM group, *n* = 24).

Subjects in both groups rated the food odors as significantly more pleasant than the odorless air (SHAM group: *t*_(22)_ = 11.62, *p* = 7.38 × 10^−11^; STIM group: *t*_(23)_ = 12.97, *p* = 4.59 × 10^−12^; Fig. 2*A*), demonstrating that food odors were perceived as rewarding. Importantly, there were no differences in the pleasantness ratings between groups (main effect of group: χ^2^(1) = 2.49, *p* = 0.115; group by odor interaction: χ^2^(1) = 1.34, *p* = 0.247). During conditioning, the percentage of trials in which participants expected a food odor after cue B increased across time relative to cue D [three-way (group × time × cue) generalized linear mixed model; main effect of cue, χ^2^(1) = 1736, *p* < 2.2 × 10^−16^; main effect of time, χ^2^(2) = 0.98, *p* = 0.613; cue × time interaction, χ^2^(2) = 254.22, *p* < 2.2 × 10^−16^; Fig. 2*B*]. There were no significant differences between groups in learning across time (main effect of group, χ^2^(1) = 0.096, *p* = 0.757; cue × group interaction, χ^2^(1) = 3.22, *p* = 0.072; time × group interaction, χ^2^(2) = 2.88, *p* = 0.24; cue × time × group interaction, χ^2^(2) = 0.36, *p* = 0.834). Most importantly, performance in the last conditioning run did not differ between groups (*t*_(45)_ = 0.0045, *p* = 0.996), demonstrating that subjects in both groups learned the associations between the cues and their associated outcomes equally well.

### OFC-targeted cTBS disrupts inference-based responding

After conditioning and immediately before the probe test, we applied 40 s of cTBS to a site in right LPFC that was individually selected to have maximal rsfMRI connectivity with the central-lateral OFC, following previously established procedures (Howard et al., 2020). Specifically, stimulation was administered in the STIM group at a high intensity that we have previously shown disrupts OFC network activity and adaptive behavior in the reinforcer devaluation task. Stimulation in the SHAM group was administered at a low intensity that was not expected to produce any impact on neural function (Howard et al., 2020).

**Figure 3.**
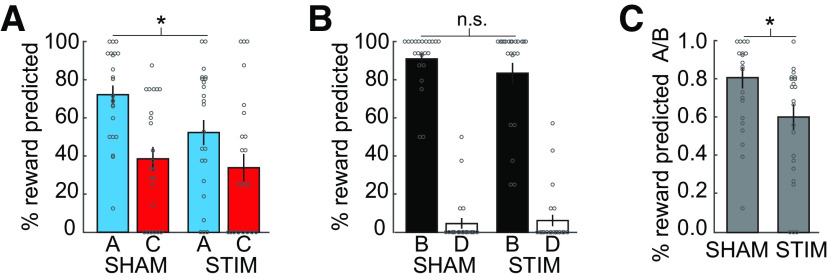
Responses based on inferred outcomes are disrupted by OFC-targeted cTBS. ***A***, The percentage of trials in which participants predicted a reward for cue A was significantly larger in the SHAM group compared with the STIM group (*p* = 0.020). There was no difference in reward predictions for cue C (*p* = 0.642). ***B***, There was no group difference in responding to cue B (*p* = 0.245) or D (*p* = 0.740). ***C***, Responses to cue A relative to cue B (A/B) were significantly stronger in the SHAM group compared with the STIM group (*p* = 0.024). Circles depict individual subjects, error bars depict SEM (SHAM group, *n* = 23; STIM group, *n* = 24). *p < 0.05; n.s., not significant.

We hypothesized that targeting the lateral OFC network with cTBS would selectively disrupt reward expectations based on inference but not those based on direct experience. In line with this, we found a significant interaction between cue type and group (χ^2^(1) = 4.95, *p* = 0.026), indicating that responses to cues A and B were differentially affected by OFC-targeted cTBS compared with the SHAM group. Indeed, follow-up *t* tests showed that this interaction was driven by significantly reduced responses to cue A in the STIM group relative to the SHAM group (*t*_(45)_ = 2.40, *p* = 0.020; Fig. 3*A*), whereas there was no significant group difference in responding to cue B (*t*_(45)_ = 1.18, *p* = 0.245; Fig. 3*B*). These results were confirmed using permutation tests (group difference in responding to A, *p* = 0.012; group difference in responding to B, *p* = 0.127). This demonstrates that the effects of OFC-targeted cTBS were specific for inference-based responding.

To obtain a more fine-grained picture of the effects of cTBS on behavior, we further analyzed response times to cues A and B. Responses to cue A were significantly faster in the STIM group compared with the SHAM group (STIM group: 1828 ± 68 ms; SHAM group: 1998 ± 56 ms; *t*_(45)_ = 1.88, *p* = 0.037, one-tailed), but there was no evidence for such a difference for cue B (STIM group: 1562 ± 51 ms; SHAM group: 1648 ± 38 ms; *t*_(45)_ = 1.33, *p* = 0.191). However, although these results are consistent with the findings reported above and suggest that the effects of OFC-targeted cTBS are selective for behavior based on inference, the cue × group interaction was not significant (χ^2^(1) = 3.24, *p* = 0.072; main effect of group: χ^2^(1) = 4.06, *p* = 0.044; main effect of cue: χ^2^(1) = 153.74, *p* < 2.2 × 10^−16^).

Reward-related responding to cue A depends not only on the ability to make an inference, but also on knowledge about the reward predicted by cue B, which was acquired through direct experience (B → reward). To further examine the effects of OFC-targeted cTBS on inference-based behavior independent of potential effects on direct experience, we normalized responses to cue A by responses to cue B. The resulting ratio (i.e., A/B) reflects the ability to infer outcomes relative to the knowledge about the directly experienced cue–reward association. This ratio was significantly smaller in the STIM group compared with the SHAM group (*t*_(45)_ = 2.33 *p* = 0.024; Fig. 3*C*). We confirmed the statistical significance of this difference using a permutation test (*p* = 0.013). Together, these results demonstrate that OFC-targeted cTBS selectively impairs behavior based on inferred outcomes but does not disrupt behavior that can be based on directly experienced outcomes.

### OFC-targeted cTBS does not disrupt memory for cue–cue associations

**Figure 4.**
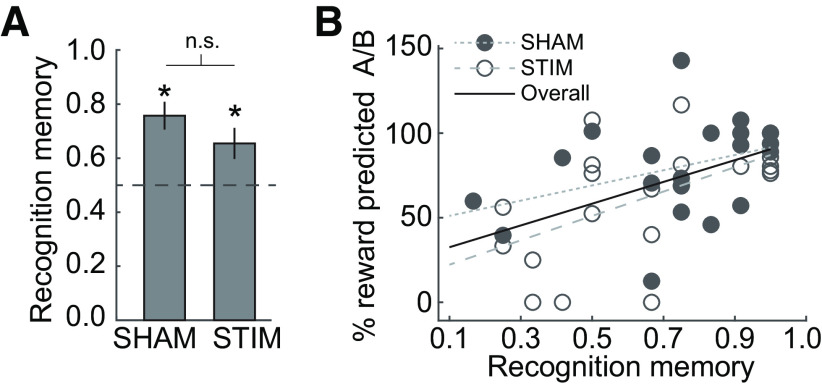
Memory for cue–cue associations and its relation to inference-based behavior is not altered by OFC-targeted cTBS. ***A***, Recognition memory for cue–cue pairs does not differ between groups (*p* = 0.188). Error bars depict SEM (SHAM group, *n* = 22; STIM group, *n* = 21). *p < 0.05; n.s., not significant. ***B***, Recognition memory for cue–cue associations was significantly correlated with responding to preconditioned cues (reward prediction responses to A/B) during the probe test (*r* = 0.51, *p* < 0.001; solid circles, SHAM group; empty circles, STIM group), and this correlation did not differ between groups (*Z* = −0.93, *p* = 0.178).

Inference also depends on memory of the cue–cue associations learned during preconditioning (Wang et al., 2020). It is therefore possible that the findings reported above reflect a failure of memory rather than inference. Although this is unlikely given that the memory of directly experienced cue–reward associations was unimpaired in the STIM group, we measured recognition memory for cue–cue associations after the probe test to rule out this potential explanation. Importantly, this memory test was still within the 50 min of presumed TMS effects. In both groups, recognition memory was significantly above chance (SHAM group: *t*_(21)_ = 5.01, *p* < 0.001; STIM group: *t*_(20)_ = 2.70, *p* = 0.013), and there was no difference between groups (*t*_(41)_ = 1.34, *p* = 0.188, permutation test, *p* = 0.129; Fig. 4*A*). Moreover, as in our previous study (Wang et al., 2020), recognition memory was significantly correlated with inference-based responding (*r* = 0.51, *p* = 0.0005; Fig. 4*B*). These correlations were significant within each group (SHAM group: *r* = 0.38, *p* = 0.039, one-tailed; STIM group: *r* = 0.55, *p* = 0.01) and did not differ between groups (*Z* = −0.93, *p* = 0.178). Of note, our between-subject design does not allow us to test whether OFC-targeted cTBS affected inference-based responding through (nonsignificant) effects on cue–cue memory. This would have required a within-subject design involving repeated measures of memory and inference with and without OFC-targeted cTBS from every participant. Together, these findings demonstrate that similar to directly experienced cue–reward associations, OFC-targeted cTBS did not significantly impair memory for cue–cue associations, or how they were used for inference-based behavior.

## Discussion

The current study shows that targeting the human OFC with network-based cTBS impairs reward-related behaviors when outcome expectations need to be mentally simulated, but not when expectations can be based on direct experience. This closely parallels previous findings from rats (Jones et al., 2012), providing converging cross-species evidence for a role of OFC networks in model-based but not model-free behavior.

As such, our findings suggest that the contribution of OFC to decision-making may be limited to situations that require model-based planning, and that choices based on direct experience may rely on value computations in other areas, such as the amygdala or striatum (Paton et al., 2006; Cox and Witten, 2019). This proposal is seemingly at odds with the large number of studies across different species that consistently report neural correlates of both inferred and directly experienced value in OFC (Hare et al., 2009; Schoenbaum et al., 2009; Barron et al., 2013; Stalnaker et al., 2014; Howard et al., 2015; Padoa-Schioppa and Conen, 2017; Suzuki et al., 2017; Klein-Flügge et al., 2019; Lopez-Persem et al., 2020; Wang et al., 2020). Why would OFC represent value signals if they are not required for behavior? One potential answer is that OFC computes and represents inferred values in all situations, even when direct experience is available, and that these signals may bias choices at any point (Ballesta et al., 2020). However, if direct experience is available, these signals are typically indistinguishable from, and redundant with, cached values represented elsewhere in the brain, such that disruption of OFC does not affect observed behavior. In contrast, because the contribution of OFC to computing model-based values is critical, disrupting its function impacts behavior when outcomes must be inferred. This proposal would explain why animals and humans with compromised OFC function are capable of making choices, but that these choices reflect previously learned values even if they are no longer valid (Gallagher et al., 1999; Izquierdo et al., 2004; West et al., 2011; Rudebeck et al., 2013; Murray et al., 2015; Gardner et al., 2017, 2018; Reber et al., 2017; Parkes et al., 2018; Howard et al., 2020).

In line with our previous work showing neural correlates of inferred outcomes in OFC (Wang et al., 2020), the current findings suggest that OFC networks are directly involved in stepping through the cue–cue and cue–reward associations when inferring outcomes at the time of decision-making. However, alternative explanations have been proposed that do not require inference at this time point. For instance, cue A could be reactivated at the time of conditioning, such that it also acquires model-free value, just like cue B. Several studies have provided correlative evidence for such mediated learning processes in areas of the medial prefrontal cortex and temporal lobe (Shohamy and Wagner, 2008; Wimmer and Shohamy, 2012; Zeithamova et al., 2012; Kurth-Nelson et al., 2015). However, it is important to note the methodological differences between these studies and ours, which may make mediated learning more or less likely. Instead of meaningful stimuli such as faces, body parts, and scenes, we used abstract symbols as cues, which are more difficult to memorize and thus perhaps less likely to be reactivated during conditioning. In addition, we explicitly instructed participants to learn the cue–cue pairings during preconditioning and to use these associations to infer outcomes in the probe test, which may have facilitated the use of inference. However, the explicit instruction may not be critical since animal studies show similar degrees of responding (Jones et al., 2012; Sadacca et al., 2016; Sharpe et al., 2017; Hart et al., 2020).

At a conceptual level, compared with just-in-time simulation of outcomes, it seems rather inefficient to engage in mediated learning for all previously experienced associations when encountering a cue. This may thus not be a general mechanism that is used in all situations. In support of this contention, preconditioned cues do not support conditioned reinforcement (Sharpe et al., 2017) and responding to these cues is sensitive to reward devaluation (Hart et al., 2020). These two behaviors are the gold standards for assessing model-free and model-based value, respectively. Moreover, pharmacological inactivation of the OFC in the probe test selectively disrupts responding to cue A without affecting responding to cue B (Jones et al., 2012). If responding to both A and B were based on the same neural mechanisms involving model-free values, then presumably the two would not be differentially affected by OFC inactivation in the final probe test in this earlier experiment or, indeed, in the current study.

However, it is important to keep in mind that behavior can be driven by several independent mechanisms and that inference-based behavior supported by mechanisms in OFC may occur in parallel with support from additional mechanisms such as mediated learning (Schlichting and Preston, 2015), which may recruit hippocampus (Shohamy and Wagner, 2008; Wimmer and Shohamy, 2012; Kurth-Nelson et al., 2015) and perirhinal cortex (Wong et al., 2019). Nevertheless, the susceptibility of inference-based responding to OFC-targeted cTBS indicates that at least some amount of behavior in our task is based on real-time model-based computations. In this regard, it is important to note that whereas OFC-targeted cTBS reduced subjects' ability to make inference-based decisions, it did not fully abolish this function. This could be related to the fact that we only applied unilateral stimulation, and thus the contralateral OFC network may have remained unimpaired. Alternatively, the remaining performance could be driven by mediated learning processes mentioned above, dependent on areas not impacted by our OFC-targeted manipulation.

It is also important to note that we did not target OFC directly. Instead, we used an OFC network-targeted approach by selecting stimulation sites in LPFC that have maximal connectivity with the central-lateral OFC, as we have done previously (Howard et al., 2020). In this previous study, the effects of cTBS were selective for the targeted central-lateral OFC network and were not observed in the medial OFC network. In addition, outside of OFC, only a few voxels in the left precentral gyrus and the left inferior frontal gyrus showed reduced connectivity. However, because the current study did not measure rsfMRI directly after TMS, we are not able to confirm that this was the case in our current sample. It is therefore possible that local effects of our stimulation on LPFC drove the observed effects. We think this is unlikely for the following reasons. First, our TMS protocol was identical to our previous study in which we did not observe any effects on LPFC activity (Howard et al., 2020). Second, our results parallel previous findings with pharmacological inactivation of OFC in animals (Jones et al., 2012). Third, although medial PFC networks have been implicated in inference processes (Zeithamova et al., 2012; Schlichting et al., 2015; Schlichting and Preston, 2015), we are not aware of similar findings related to LPFC. However, cTBS could have affected reliability signals in LPFC that have been shown to correlate with the arbitration of behavioral control between model-based and model-free processes (Lee et al., 2014).

An additional limitation is our sham condition, which involved stimulation at 5% MT. This is noticeably different from stimulation at 80% MT in terms of auditory and somatosensory stimulation (i.e., discomfort at the scalp and facial muscle twitches). These unintended peripheral effects of TMS could have driven the observed behavioral effects, rather than the neural changes induced by cTBS. Although this concern could be better addressed by additional control groups involving cTBS over parts of cortex that are not part of the targeted network, we believe peripheral effects are unlikely to be the cause of the behavioral impairment for two reasons. First, the effects of cTBS were specific to inference-based behavior, and no differences were found for behavioral responses based on direct experience or memory for cue–cue associations. It is difficult to conceive why the peripheral effects of the TMS would have highly disparate effects on two almost identical behaviors that only differ in their requirement for inference. Second, our previous study using OFC-targeted TMS involved an additional control condition that was matched for somatosensory stimulation (Howard et al., 2020). Despite comparable peripheral effects, behavioral and neural effects in this control condition differed significantly from active cTBS but were similar to the 5% sham condition. We therefore think it is unlikely that our results were driven by unintended non-neuronal effects of cTBS.

In summary, our results support the idea that human OFC networks are necessary for inference-based behavior, whereas they are not critical to support decision-making when direct experience is available. Deficits in decision-making and altered OFC function are hallmarks of many neuropsychiatric disorders, including substance use disorder (Zilverstand et al., 2018) and obsessive-compulsive disorder (Menzies et al., 2008). Our findings may offer a conceptual framework for understanding how OFC dysfunction may disrupt behavior in these conditions.
